# Itk: The Rheostat of the T Cell Response

**DOI:** 10.1155/2011/297868

**Published:** 2011-04-07

**Authors:** Juris A. Grasis, Constantine D. Tsoukas

**Affiliations:** Department of Biology, San Diego State University, San Diego, CA 92182-4614, USA

## Abstract

The nonreceptor tyrosine kinase Itk plays a key role in TCR-initiated signaling that directly and significantly affects the regulation of PLC*γ*1 and the consequent mobilization of Ca^2+^. Itk also participates in the regulation of cytoskeletal reorganization as well as cellular adhesion, which is necessary for a productive T cell response. The functional cellular outcome of these molecular regulations by Itk renders it an important mediator of T cell development and differentiation. This paper encompasses the structure of Itk, the signaling parameters leading to Itk activation, and Itk effects on molecular pathways resulting in functional cellular outcomes. The incorporation of these factors persuades one to believe that Itk serves as a modulator, or rheostat, critically fine-tuning the T cell response.

## 1. Introduction

Normal T lymphocyte activation occurs through its antigen receptor leading to various signaling cascades and ending in a certain function. These activating signaling cascades are exquisitely balanced, as different signals can lead to many different functional outcomes. Inappropriate signaling or skewed signals can cause many problems for the T cell and for a functional immune system. Aberrant T cell function can induce many devastating diseases such as leukemia and autoimmune disorders.

**Figure 1:**
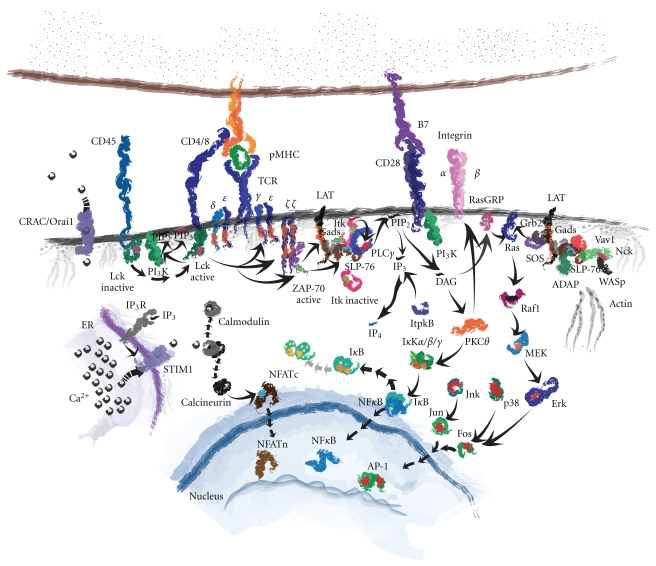
*T cell receptor activated signal transduction pathways.* Cartoon diagram of the critical protein interactions necessary for the activation of a T cell when engaging an antigen-presenting cell.

When a T cell encounters an antigen-presenting cell (APC), it must be able to discriminate whether or not the APC is functional. This occurs through physical interaction between surface molecules of the APC and T cell, translating that information into a response. This requires many coordinated molecular interactions from the cell surface, through the cytoplasm, on to the nucleus, and in some instances, back out through the cell surface again [1]. An illustration of this is shown in Figure 1. At the cell surface, interaction between the peptide-loaded major histocompatibility complex (pMHC) of class II or class I on the APC with the T cell receptor (TCR) on the T cell coordinates with coreceptor binding of CD4 on helper T cells or CD8 on cytotoxic T cells, respectively. This interaction causes the dissociation and activation of the tyrosine phosphatase CD45 from CD4/CD8, which dephosphorylates CD4/CD8 bound Src kinase Lck on its inhibitory tyrosine (tyrosine 505). With activated Lck in tow, CD4/CD8 co-receptor pulls Lck into proximity of the TCR/CD3 complex. Lck then phosphorylates the intracellular tyrosine activation motifs (ITAMs) within the CD3 complex. Phosphorylation of the CD3 ITAMs promotes the docking of other molecules to the CD3 complex, namely the zeta-associated protein of 70 kilodaltons (ZAP-70) which is also phosphorylated by Lck for its activation [2, 3]. Activated ZAP-70 then phosphorylates the linker for activated T cells, or LAT, a transmembrane and palmitoylated adaptor that bridges the initial TCR signal to many downstream signaling events [4].

Phosphorylation of LAT is paramount for subsequent signaling due to the critical nature of the proteins binding to it [5]. Another cytoplasmic adaptor molecule, the SH2 domain containing leukocyte phosphoprotein of 76 kilodaltons (SLP-76), binds to LAT through Gads where it becomes phosphorylated by ZAP-70 [6]. SLP-76 binds to and inducibly recruits many other cytoplasmic molecules to the activated LAT complex, including PLC*γ*1, ADAP, Nck, and Itk, the subject of this paper [7]. LAT-recruited Itk becomes transphosphorylated at the membrane by Lck on its activation tyrosine (tyrosine 511), and then autophosphorylates itself on tyrosine 180 for full activation [8]. Activated Itk phosphorylates PLC*γ*1 on tyrosines 775 and 783 leading to the activation of this lipase [9–11]. 

Itk-activated PLC*γ*1 cleaves membrane phosphatidyl inositol (4,5) bisphosphate (PIP_2_) into two secondary messengers, inositol 1,4,5-trisphosphate (IP_3_) and diacylglycerol (DAG). IP_3_ diffuses into the cytoplasm to bind its cognate IP_3_ receptors (IP_3_R) on the endoplasmic reticulum (ER) leading to the release of calcium in the cytoplasm [12]. Cytoplasmic Ca^2+^ binds calmodulin, causing its inhibitory release from calcineurin, freeing this phosphatase to dephosphorylate cytoplasmic localized nuclear factor of activated T cells (NFAT). NFAT then translocates into the nucleus to perform its transcription factor duties of genetic regulation [13]. The other PLC*γ*1-generated secondary messenger, DAG, activates protein kinase C theta (PKC*θ*), which leads to the release of the nuclear factor of kappa light chain enhancer in B cells (NF*κ*B) into the nucleus to perform its transcription factor duties [14, 15]. DAG also promotes the phosphorylation of Ras guanyl releasing protein (RasGRP), a GTP exchange factor (GEF) that activates Ras, leading to the downstream activation of mitogen-activated protein kinase (MAPK) pathways [16], the MAPKs jun-N-terminal kinase (JNK) and p38 phosphorylate Jun and Fos, respectively, prompting them to translocate into the nucleus and combine to form the AP-1 transcription factor important for genetic regulation [17].

The TCR-activated, LAT-bridged, SLP-76 signalosome brings in two more players important for the sustained activation of the T cell necessary for its full response. These two molecules are the adhesion- and degranulation-promoting adaptor protein (ADAP) and noncatalytic region of tyrosine kinase (Nck). ADAP is an adaptor protein that leads to the intracellular activation of extracellular integrin adhesion molecules, in a process termed as “inside-out” signaling [18–20]. Integrin activation helps to maintain T cell contact with the APC for an extended period to allow full T cell response to the presented antigen [21]. Nck is an adaptor that brings the cytoskeleton organizing molecule, Wiskott-Aldrich syndrome protein (WASp), to the proximity of the SLP-76 signalosome [22, 23]. WASp is indirectly activated through the GEF Vav1. Vav1 exchanges GTP for GDP for Rho GTPases, particularly that of Cdc42, which in the GTP-bound conformation binds to and activates WASp. This causes WASp to destabilize its autoinhibitory condition and take on an open conformation allowing the Arp2/3 complex to bind and seed actin polymerization [24]. Therefore, active WASp enables actin to be reorganized at the T cell junction adjacent to the APC, causing the T cell to stay stably in contact with the APC for the duration of its response, a process necessitated by Itk.

The modulator of this TCR-mediated response is Itk, and as such, the focus of this paper will be on Itk, an enigmatic kinase that serves as a rheostat for many of the signaling pathways needed for a full and effective T cell response. Itk is needed for the regulation of Ca^2+^ flux, actin polymerization, integrin binding, and transcriptional activation. Consequently, it has a profound role in the development of T cells, cytokine expression, and the clearance of pathogens. The mention of other lymphoid signal transduction molecules, particularly that of other Tec family kinases, will only be alluded to as a reference to Itk in order to highlight the importance of Itk [25, 26]. 

## 2. Identification of Itk

Interleukin-2 inducible tyrosine kinase was discovered and cloned by three independent research groups. Stephen Desiderio's group was the first to identify the gene and named it Itk (IL-2 Inducible Tyrosine Kinase) [27]. This was quickly followed by Leslie Berg's group (T-cell-specific tyrosine kinase, Tsk) [28] and Toshiaki Kawakami's group (expressed in mast and T cells, emt) [29]. This nearly simultaneous cloning of the gene and subsequent naming by three different groups led to some conflicting nomenclature initially, but the gene name is now accepted as Itk. This kinase is a 72-kDa protein expressed in the thymus, spleen, and lymph nodes. More specifically, Itk is expressed in T cells, natural killer cells, natural killer T cells, and mast cells. It is chromosomally localized to 5q31-32 in humans [30]. This localization is unique to Itk as other Tec family kinases are seemingly duplicated; Tec and resting lymphocyte kinase (Rlk) both are on the 4p12 locus [31], while Bruton's tyrosine kinase (Btk) and bone marrow kinase (Bmx) are both on the X chromosome (albeit separated somewhat, Btk on Xq22 and Bmx on Xp22 [32]). Evolutionarily, the Tec kinases have been found in flies (*Drosophila melanogaster*), zebrafish (*Danio rerio*), skates (*Raja eglanteria*), and in sea urchins (*Anthocidaris crassispina*) [33]. Two different forms of Itk have been cloned in the mouse, which have six amino acids either included or deleted [34]. The shorter version is exclusively detected in human cells. Itk has substantial effects on T cell development and function, due in large part to its disproportionate expression levels compared to other Tec kinases. As measured by qRT-PCR, naïve mouse T cells contain 100-fold more copies of Itk compared to Tec, and 3-fold more copies than that of Rlk [35]. Upon T cell stimulation, Itk expression is increased, especially in T_H_2 cells, while Rlk expression is reduced and becomes relegated to T_H_1 cells [36]. Tec expression in T cells increases only after several days of stimulation [37].

## 3. Protein Structure of Itk

Itk is a modular protein, consisting of five distinct structural domains [38]. At its amino terminus, Itk contains a pleckstrin homology (PH) domain, which allows the protein to bind phosphorylated lipids on the membrane. Adjacent to this is the Tec homology (TH) domain from which comes Itk's familial association. This domain contains a polyproline rich region (PRR) necessary for binding Src homology 3 (SH3) domains. The adjoining domain is a SH3 domain, which binds to PRR both in cis- (to itself) and in trans- (to other proteins). The following Src homology 2 (SH2) domain enables the protein to bind tyrosine-phosphorylated substrates. At its carboxyl terminus, Itk possesses an Src homology 1 (SH1) or kinase domain, which specifically phosphorylates tyrosine residues, particularly that of PLC*γ*1 [39]. Itk, as well as the Tec kinases, shares evolutionary similarities to the Src family kinases in structure. However, the Tec kinases lack the N-terminal myristoylation sequence constitutively localizing Src kinases to the membrane and lack the C-terminal negative regulatory tyrosine residue present on Src kinases.

### 3.1. The Pleckstrin Homology (PH) Domain (Amino Acids 5–112)

PH domains are protein-lipid interaction domains of about 100 amino acids in size, forming a *β*-barrel from two *β*-sheets and a carboxyl-terminal *α*-helix [40, 41]. A unique feature of the Tec family tyrosine kinases is the presence of a PH domain. Rlk is an exception as it contains a cysteine palmitoylation motif and no PH domain [42]. The PH domain contains the propensity to bind phospholipids in many forms, including phosphatidylinositol-3,4,5 triphosphate (PIP_3_), PI(4,5)P_2_, and PI(4)P, with binding of Itk to PIP_3_ at highest affinity. Itk binding to the plasma membrane through its PH domain is induced through the activity of PI_3_K, which phosphorylates PIP_2_ to PIP_3_. Addition of PI_3_K inhibitors prevents Itk from localizing to the plasma membrane and becoming active [43–45]. Therefore, Itk is targeted to the membrane and activated through a PI_3_K-dependent mechanism. Interestingly, localization to the plasma membrane through the PH domain is not enough for Itk's activation. Pleckstrin homolgy domain deleted Itk replaced with a myristoylation sequence enables Itk to localize to the membrane, but it does not become tyrosine phosphorylated upon TCR stimulation [43]. These data indicate that the PH domain contains some other role vital to Itk's activation other than simple membrane localization. PH domain binding to PIP_3_ localizes Itk in proximity of its activating kinase, Lck, thereby increasing Itk's kinase activity [43, 44, 46]. Also, this domain is integral for binding to the nuclear membrane when the molecule is shuttled to the nucleus [47]. Another unique feature of the Tec kinases is the presence of a PH domain FYF motif. This motif in Btk is linked to agammaglobulinaemia in humans; however, this motif's function has not yet been addressed in Itk. Although selective PH domain inhibitors of Btk have been reported, such as the quinone epoxide terreic acid [48], no selective PH domain inhibitors of Itk have been reported. Recently, the PH domain of Itk was shown to bind to inositol-1,3,4,5-tetrakisphophate (IP_4_) *in vitro* with a functional *in vivo* consequence [49]. Molecularly, this causes Itk's disruption of its inducible localization to the T cell/APC contact site and for its activation. Cellularly, this creates a phenotype where thymocytes undergoing positive selection become unable to progress beyond the CD4^+^CD8^+^ double positive stage of development. This necessitates Itk's involvement in T cell development, which requires an alternative role for Itk's PH domain. These combined data suggest an intriguing role of the PH domain of Itk both in its molecular activation and Itk's effect on cellular function.

### 3.2. The Tec Homology (TH) Domain (a.a. 115–163)

Itk's TH domain contains a familial Tec motif of a conserved 27 a.a. sequence at the amino-terminus of the domain [50] as well as a proline rich region (PRR) at a.a. 153–163. This domain is integral to the activity of Itk as it is thought that this domain binds to its own SH3 domain in an autoinhibited state [51]. Disruption of this association, either by competitive interaction with another peptide sequence or by mutation, is surmised to relieve this autoinhibition and activate the kinase. However, deletion of only the PRR has an opposite effect, it reduces Itk's basal activity by 50% [52]. Additionally, it has been reported that this domain contains a familial Zn^++^ binding motif, which is critical for oligomerization of Itk at the cell surface [53]. As of yet, no protein has been shown to functionally bind to the TH domain of Itk. Proteins shown to bind *in vitro* have later been shown to bind indirectly *in vivo*, either through another Itk structural domain or through another molecule entirely [54]. Given the paradoxical nature between the structure and the function of this domain, more work should be done to identify what the TH domain is doing in Itk.

### 3.3. The Src Homology 3 (SH3) Domain (a.a. 174–230)

SH3 domains allow for protein-protein interactions to proline peptide sequences (either (R/K)XXPXXP or PXXPXR motifs, where X is any amino acid) facilitating protein function or cellular localization. Not only is the SH3 domain of Itk involved with the binding of internal and external PRRs, but it is also involved in the negative regulation of its own catalytic activity. For example, elimination of the SH3 domain or mutation of a Tec family conserved tryptophan (W208K) that is required for SH3 binding to PRR causes a spontaneous activation of the enzyme, presumably through the release of autoinhibition [55]. Paradoxically, auto-phosphorylation on tyrosine 180 leads to the activation of Itk [8, 56], so elimination of this domain should theoretically lead to an inactive condition. However, elimination of this domain and mutational disruption most likely leads to a conformational release of autoinhibition, allowing the kinase domain to become exposed and catalytically active. It is also possible that the phosphorylation within this domain prevents the binding of a repressor protein though no such protein has been found. There are a plethora of proteins that bind the SH3 domain of Itk *in vitro* [57], including WASp, Sam68, Cbl, SLP-76, and Vav1, many of which have not been confirmed *in vivo*. One exception is the recent report of the *in vivo* interaction of Itk with SLP-76 [58]. Utilizing a cell-permeable peptide to competitively inhibit the Itk-SH3 interaction with SLP-76 PRR, Itk activity and functional T cell response was affected, indicating an important role of the Itk-SH3 domain interaction with SLP-76. The interaction between Itk's SH3 domain and the nuclear import chaperone karyopherin *α* (Rag cohort 1*α*, Rch1*α*) leads to Itk's transport into the nucleus [47]. This interaction requires Rch1*α*'s proline 242, as a mutant Rch1*α* (P242A) decreased nuclear localization of Itk and diminished IL-2 production. As described later, this is important as Itk not only serves as a cellular membrane proximal kinase linking early signal transduction events, but it also serves to regulate transcriptional activation of certain proteins in the nucleus.

### 3.4. The Src Homology 2 (SH2) Domain (a.a. 237–329)

SH2 domains allow for inducible protein-protein interactions with phosphotyrosine-containing peptide sequences [59, 60]. Itk's binding to tyrosine phosphorylated substrates not only provides the mechanism for it to inducibly interact with protein partners, but it has shined light on its own localization and consequent activation. Mutation of the SH2 domain of Itk, either through deletion or point mutation of the tyrosine binding pocket, leads to the inactivation of Itk [55]. Itk has been reported to bind to SLP-76 [54, 61], LAT, [44, 55], and Vav1 [62] through its SH2 domain, although it has not yet been determined to which of these molecules the SH2 domain of Itk has a higher affinity. Itk also binds to PLC*γ*1 through its SH2 domain, though it is not resolved as to whether it is direct [39], or indirect through SLP-76 [9, 63]. Importantly, it has been shown that Itk is negatively regulated through its SH2 domain by the prolyl-isomerase cyclophilin A (CypA), whose binding to Itk can be competed against by Cyclosporin A leading to Itk's activation [64]. Peptidylprolyl isomerases, such as CypA, convert proline peptide bonds to cause a cis- or trans-conformational switch in the protein with which it interacts. CypA binds to a proline residue in the SH2 domain of Itk (P287), promoting the cis-intramolecular interaction with the SH3 domain. This proline residue switch is unique to Itk, as it is not a shared phenomenon with the other Tec kinases [65]. When CypA is relieved of binding to Itk, an isomeric switch within the SH2 domain causes Itk to take on a transconformation, allowing it to bind to other proteins. These data imply that CypA is a negative regulator of Itk activity. Consistent with this idea, T cells deficient for CypA display an overall pattern of increased signaling in response to TCR stimulation, with an increase of PLC*γ*1 phosphorylation in particular [66]. Given the inducible relieving of repression and consequent functional binding to many key signaling molecules, the SH2 domain of Itk is of vital importance for its activity.

### 3.5. The Src Homology 1 (SH1) Domain (a.a. 363–612)

The sting for Itk's kinase activity resides within its SH1 domain. Herein lies its ATP binding pocket, allowing it to enzymatically transfer terminal ATP phosphate to its protein target(s). Also within this domain is tyrosine 511 which needs to become transphosphorylated by Lck for the initial activation of Itk [67]. X-ray crystal structures of the kinase domain of Itk have been resolved both in the non-phosphorylated and in the phosphorylated states [68]. Interestingly, phosphorylation of tyrosine 511 did not induce a conformational change in this domain indicating that although phosphorylation of this residue is required for the activity of this enzyme, it does not do so through a phosphorylation-dependent conformational switch. Rather, phosphorylation of the kinase domain could lead to some secondary function. Further, the isolated kinase domain of Itk is similar to the other Tec kinases in that they are kinetically inactive [69]. This is in stark contrast to any other SH1 containing kinase. This indicates that other domains of Itk help to regulate the kinase. Finally, the known targets of Itk kinase domain transphosphorylation are PLC*γ*1 tyrosines 775 and 783 [63], T-bet tyrosine 525 [70], Tim-3 tyrosine 265 [71], and TFII-I tyrosine 248 [72].

## 4. Upstream Regulators of Itk

**Figure 2:**
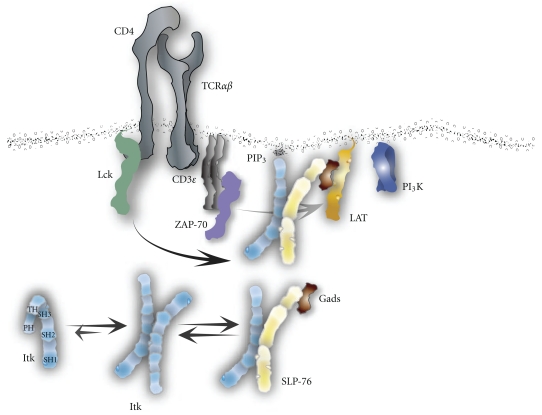
*Mode of Itk activation through the T cell receptor. *While residing in the cytoplasm, Itk preferentially takes on an inhibited homodimer conformation and in some instances as an inhibited independent cis-conformation. Upon T cell engagement, Itk is able to bind SLP-76 through its SH3 domain and shuttled to the LAT signalosome. Once there, activated ZAP-70 phosphorylates SLP-76 on tyrosine 145 which Itk then binds to with its SH2 domain to solidify the connection at the signalosome. At which time Itk localizes to the plasma membrane by binding PI_3_K-generated PIP_3_ with its PH domain and Itk then becomes tyrosine phosphorylated by Lck on tyrosine 511. This leads to Itk's catalytic activity that first phosphorylates itself on tyrosine 180 culminating in a fully activated enzyme.

The activation of Itk is a complex orchestration of events (Figure 2). This kinase is activated through a myriad of surface receptors, including the TCR/CD3 signaling complex, co-receptors, chemokine receptors, and heterotrimeric G-protein-coupled receptors (GPCRs). Prior to the activation of the T cell, Itk resides in the cytoplasm in a closed, autoinhibited state. This inhibitory conformation occurs through the cisbinding of its SH3 domain to the PRR of the TH domain [51]. More recently, however, it was found that the SH2 and SH3 domains of Itk could dimerize with each other in a trans-head-to-tail manner that could preclude or mitigate the cis-binding of a single Itk molecule to itself [73]. Further, it was found that full-length Itk self-associates intermolecularly [74]. This intermolecular clustering negatively regulates Itk and, once disrupted, leads to Itk activity. Continuing to determine the conformational state and activation of full-length Itk is of critical importance, as the information will be utilized for inhibitor development.

Itk associates with SLP-76 while in the cytoplasm, and is shuttled to the membrane-associated LAT signalosome by SLP-76 upon TCR/CD3 stimulation [54]. While at the LAT signalosome, Itk becomes phosphorylated at tyrosine 511 of the SH1 domain by Lck [8, 67]. Transphosphorylation activates Itk kinase activity, whereupon the kinase autophosphorylates itself at tyrosine 180 of the SH3 domain [8]. Cis-phosphorylation fully opens the protein, allowing the PH domain to bind PI_3_K phosphorylated lipids at the membrane and therefore acts as a fully active kinase within the LAT signalosome. Cells lacking Lck or containing a mutant Itk phenylalanine substitution for tyrosine at the transphosphorylation position 511 leads to a kinetically inactive Itk [67]. Further, mutation of Itk at the auto-phosphorylation site tyrosine 180 to phenylalanine impairs Itk's activity in response to stimulation [8]. This Itk auto-phosphorylated tyrosine lies within the substrate binding sequence of the SH3 domain [51], leading one to speculate that this tyrosine regulatory site is not just important for enzymatic activity, but for protein-protein interactions as well. Similarly, Btk's autophosphorylation site at tyrosine 223 only binds to WASp while in the non-phosphorylated state, but only binds to Syk while in the phosphorylated state [75]. It would be reasonable to speculate that Itk acts in a similar fashion.

### 4.1. T Cell Receptor Activation

TCR engagement leads to the pivotal activation of two molecules that are integrally important for the activation of Itk, Lck, and PI_3_K. Activation of Lck through the dephosphorylation of its inhibitory tyrosine by CD45 causes a rapid phosphorylation of receptor components as well as associating molecules such as ZAP-70 and downstream adaptor molecules LAT and SLP-76. Recruitment of Itk to the TCR-nucleated signalosome is mediated through SLP-76, which brings Itk from the cytoplasm to the plasma membrane where Itk PH domain can bind to PI_3_K phosphorylated phospholipids [58]. Recruitment by SLP-76 to the ZAP-70 primed LAT signalosome leads Itk to be transphosphorylated by Lck. This combination of events leads to the fully activated state of Itk, one that is relieved of autoinhibited restraints, and one that can phosphorylate downstream targets.

### 4.2. Co-Receptor Activation

Upon CD28 stimulation, Itk binds to CD28 and becomes tyrosine phosphorylated by Lck [76–78]. Itk's interaction with the cytoplasmic polyproline motif of CD28 through its SH3 domain facilitates both the activation of Itk and the tyrosine phosphorylation of CD28 on all four tyrosines found in its cytoplasmic tail [79–81]. This activation of Itk helps to amplify TCR signals, which has a profound effect on Ca^2+^ mobilization [82]. This association was originally proposed to illuminate Itk as a negative regulator of T cell signaling through CD28, as mice lacking Itk have an increased proliferative response to CD28 stimulation [83]. However, more recent findings indicate that Itk is not essential for CD28 signaling [84]. In this work, investigators used purified CD4^+^ T cells from Itk^−/−^ mice and noted that these cells already have an activated or memory phenotype that becomes enhanced upon stimulation. It was therefore found that CD28 signaling was normal in Itk^−/−^ cells and Itk does not act as a negative regulator of CD28 stimulation. Similar to the binding of Itk to CD28, Itk has been reported to bind to the PRR on the cytoplasmic portion of CD2, enabling Itk to become activated by Lck upon CD2 cross-linking [85, 86]. Itk's explicit association with these co-receptors inspire further evaluation as it relates to signal transduction pathway cross-talk.

### 4.3. Chemokine and Hetero-Trimeric G-Protein Coupled Receptors

Both sets of binding G-protein subunits can functionally associate with Itk. The G-protein *βγ* subunits have been shown to bind to Itk through Itk's PH domain and can promote Itk activity [87]. Chemokines are small proteins that are inducibly secreted by resident cells to promote the recruitment and migration of lymphocytes to an area of infection. Chemokine stimulation linking G-protein coupled receptors to actin polymerization events have shown that Itk can mobilize to the plasma membrane and become tyrosine phosphorylated upon Stromal cell-derived Factor 1-alpha (SDF-1*α*, also known as CXCL12) chemokine stimulation [88, 89]. Itk's response to SDF-1*α* is sensitive to both PI_3_K and pertussis toxin from the bacterium *Bordetella pertussis*, which inactivates *Gα*_*i*_. Additionally, overexpression of G*α*_transducin_ leads to the dominant-negative inhibition of endogenous G*βγ* subunits, which affects recruitment of Itk to the plasma membrane in HeLa cells [88]. Since PI_3_K can be activated through G*βγ*, it follows that recruitment of Itk to the plasma membrane upon chemokine stimulation is dependent on a G*βγ*-PI_3_K pathway. This was shown to be true in a recent paper detailing the T cell specific adapter protein (TSAd) promoting the migration of T cells through the interaction of the G*β* subunit, leading to CXCL12-induced Itk phosphorylation, actin polymerization, and cellular migration [90]. The Schwartzberg group also showed that mutant Itk can block the chemokine-induced polarization and activation of the Rho GTPases Cdc42 and Rac [88]. Consistent with the molecular findings, T cells from Itk^−/−^ mice are deficient in their ability to home to the lymph node, and as such, there are reduced numbers of T cells in the lymph nodes in comparison to the numbers of T cells in the spleens of these mice [88]. Further, Itk deficient T cells display an impairment in their ability to migrate to the lungs in response to CXCR4 ligand [89]. This finding has been confirmed in mice treated with a kinase-domain Itk-specific inhibitor, whose T cells were also reduced in ability to home to the lungs [91]. All of the above Itk-activating pathways coordinate to tune the immune response leading to downstream effector function.

## 5. Downstream Itk Effectors

Intriguingly, Itk is a known activator of many different downstream targets leading not only to the immediate activation of certain signal transduction pathways, but also to transcriptional activation of certain genes corresponding to the development of T cell subsets.

### 5.1. PLC*γ*1 and Calcium Mobilization

As mentioned above, Itk is a critical activator of PLC*γ*1. T cells from Itk^−/−^ mice display diminished tyrosine phosphorylation of PLC*γ*1 [92] and of tyrosine 783 in particular [63], the critical activating site for this lipase. The consequence of PLC*γ*1 inactivation is lack of downstream secondary messenger IP_3_ binding to IP_3_R preventing the release of intracellular Ca^2+^ stores [92, 93] and the inhibition of calcium release activated channels (CRAC) at the plasma membrane [93]. CRAC-mediated capacitative influx of Ca^2+^ from extracellular sources prolongs the signaling necessary for later action, including transcriptional activity. This sustained Ca^2+^ influx is necessary for long-term signaling in the T cell as well as the formation and the continued presence of the immunological synapse [94]. Accordingly, Itk-deficient T cells are severely diminished in their short-term and long-term Ca^2+^ flux abilities. The failure of sustained Ca^2+^flux leads to the downstream inactivation of Erk [92], which manifests itself into a lack of NFAT translocation to the nucleus to initiate its transcriptional activity. Kinase-inactive mutants of Itk cannot phosphorylate PLC*γ*1 and therefore, downstream activation of Erk and of TCR-induced NFAT reporter activation is compromised [54]. This inactivation is also due to the lack of PLC*γ*1 generated secondary messenger DAG, a necessary component for the activation of the DAG-binding domain containing protein RasGRP, which is required for Erk pathway activation in T cells [95, 96]. There is some functional redundancy to the Tec kinases in T cells, as PLC*γ*1 tyrosine phosphorylation, and subsequent Ca^2+^ flux is only reduced, and not ablated in cells lacking Itk, suggesting a functional role for Rlk and Tec in these processes [92, 93]. T cells from Rlk or Tec knockout mice do not display these phenotypes, as PLC*γ*1 phosphorylation and Ca^2+^ flux are intact. However, T cells from double knockouts of Itk and Rlk diminish these two characteristics even further [92]. Further, overexpression of Tec or Rlk in the absence of Itk can functionally rescue the Itk-deficient phenotype in T cells [37].

Itk has been established to regulate Ca^2+^ flux in T cells through PLC*γ*1, the lipase that directly leads to the release of Ca^2+^ from ER stores. However, this does not explain Itk effect on prolonged Ca^2+^ flux. The recently characterized CRAC channels would further explain the relationship between Itk and sustained Ca^2+^ signaling. How ER-released Ca^2+^ stores regulate CRAC channel operation was recently identified to be due to an ER-resident Ca^2+^ sensor named STIM1 [97]. STIM1 knockdown leads to a complete abrogation of store-operated calcium entry in T cells. Furthermore, upon TCR-induced activation of the T cell, STIM1 colocalizes with a plasma membrane resident CRAC channel named Orai1 at the immunological synapse, which leads to a sustained Ca^2+^ flux for the T cell [98]. Orai1 is a tetraspanin pore-forming subunit of the CRAC channel, which when absent or mutated also causes an abrogation of sustained Ca^2+^ flux [99, 100]. This mutation (R91W) in Orai1 manifests in human disease, as one aspect for the development of SCID is due to the attenuation of Ca^2+^ flux after T cell activation [101]. Given Itk's strong role in sustained Ca^2+^ flux, it would be of interest to determine whether Itk has an effect on the activity of either STIM1 or Orai1. Regardless of a direct or indirect relationship, it might still provide a more specific method for temporary therapeutic relief of some inflammatory responses, such as graft rejection in transplantation, psoriasis, arthritis, or colitis. Current therapies for these disorders involve the use of cyclosporin A and FK506 to inhibit calcineurin activity leading to reduced Ca^2+^ flux and reduced T cell activity. Unfortunately, since calcineurin is widely expressed in most cell types, these treatments often lead to unwanted and sometimes severe side effects. If one could directly affect Ca^2+^ flux in T cells using Itk inhibitors, without affecting other cells, it would be extremely beneficial for the treatment of these disorders.

### 5.2. Actin Reorganization

The Tec family kinases were initially implicated in the regulation of the actin cytoskeleton through work done in *Drosophila*. The Tec family kinase Tec29 was found to be required for the growth of ring canals, a necessary bridge between the nurse cells and oocyte during fly embryogenesis [102, 103]. The ring canals in the Tec29 mutant egg chambers failed to form correctly, thereby failing to nourish the maturing oocyte. This data shows an evolutionarily conserved function for Tec kinases in actin regulation.

Within mammalian T cells, it was discovered by two groups that Itk was involved in TCR-induced actin polymerization [104, 105]. These data showed that in T cells lacking Itk, actin directed processes (such as lamellipodial formation and directed actin polymerization) were impaired. The significance of this deficiency is striking, as the defects in actin polymerization and polarization in Itk^−/−^  T cells is complete, whereas the Itk defect in Ca^2+^ flux is only reduced. Additionally, this defect is most likely entirely due to the absence of Itk, as T cells deficient for both Itk and Rlk are just as defective in actin polymerization as Itk singly deficient T cells, suggesting no functional redundancy [105]. Further, overexpression of Itk leads to an increase in membrane ruffling (lamellipodia) [44]. This regulation of actin is through the interaction of Itk with two molecules directly involved with actin polymerization, Vav1, and WASp [62, 106]. Vav molecules are GTP exchange factors (GEFs) that exchange GDP for GTP to activate members of the Rho GTPase family, particularly that of Cdc42 in T cells. Vav1 deficient T cells not only display a defect in the ability to activate the Rho proteins, but also show very striking actin defects. These cells fail to polymerize actin, show impaired integrin adhesion and clustering, and display broader cellular defects than that of T cells lacking either Itk or WASp [107, 108]. Initially, it was determined that Itk affected Vav1 localization, as T cells deficient in Itk failed to localize Vav1 when engaging antigen-pulsed APCs [105]. This defect had a functional consequence, as activated Cdc42 no longer localized to the contact site as measured with a biosensor. Itk was discovered to constitutively associate with Vav1 [62], an association that was dependent upon Itk's SH2 domain. This interaction confirms a hypothesis that Itk has a kinase-independent function [109], thus broadening the role of this kinase beyond the simplistic idea that a kinase must only act as a kinase, that is, to phosphorylate targets. Notable, however, is the finding that Vav1 still becomes tyrosine phosphorylated in cells lacking Itk (although it has not been determined whether tyrosine 174 phosphorylation is defective, the key activating tyrosine in Vav1 [110]), a finding compounded by the data that Itk activation is also abnormal in cells lacking Vav1 [111]. Lately, Itk^−/−^Vav1^−/−^ mice were generated and were shown to be strongly reduced in the number of double-positive thymocytes and displayed impaired positive selection [112]. This increased developmental defect shows that Itk and Vav1 share some mechanistic similarities affecting T cell development as well.

The Wiskott-Aldrich Syndrome protein (WASp) is the gene product whose deficiency leads to Wiskott-Aldrich Syndrome, a fulminant state that includes lymphopenia mediated by a defect in actin regulation. WASp is vital in actin regulation as it activates the Arp2/3 complex which helps to nucleate new actin filaments [113–117]. WASp is indirectly dependent upon Vav1 for its activation as it needs to bind to GTP bound Cdc42 to release its autoinhibition and activate the Arp2/3 complex, leading to the nucleation of new actin filaments [24]. Itk has been shown to bind WASp, both *in vitro *through its SH3 domain [57] and *in vivo *using full-length molecules [106]; however, the functional outcome of this interaction is muddied because Itk seems to be required for WASp's localization [105], but not for its phosphorylation [118], a requirement necessary for WASp's full activity [119, 120]. Again, this implies a kinase-independent function for Itk's role in actin polymerization.

The intricate dance between Itk, Vav1, and WASp, as it relates to TCR-induced actin polymerization remains an interesting topic, as it seems that Itk affects both Vav1 and WASp in a kinase-independent manner. This does not, however, preclude that Itk might have a kinase-specific role for the continued activation of these molecules. If Vav1 is probed for its phosphorylation on its activating tyrosine 174 [121] and if WASp is probed for its phosphorylation on its activating tyrosine Y291 [119] in the presence or absence of Itk, one will get a better idea of Itk's role in a kinase-dependent or kinase-independent manner. Furthermore, it has been reported that the Tec family kinase Btk not only binds actin directly through its PH domain [122], but it can also tyrosine phosphorylate WASp as well [123, 124]. Also, given that the tyrosine kinase Abl can bind and phosphorylate actin directly [125], and that the tyrosine kinase Fyn can phosphorylate WASp [118], it may be useful to pursue whether Itk can perform any of these duties as well.

The effects of Itk seen on actin polymerization in T cells also dismisses the idea that the cellular defects seen in Itk^−/−^ cells is due merely to the molecular inactivation of PLC*γ*1. The decrease in actin polymerization can affect signal strength and signal duration, as well as affect integrin-mediated adhesion leading to the known defects in Itk^−/−^ T cells including altered CD4/CD8 ratios, specific decrease in cytokine production, and in the proliferation of these cells. It is likely that the defect in cytoskeletal reorganization contributes to the decreased response of Itk^−/−^ T cells as a result of inefficient immunological synapse formation [126].

### 5.3. Adaptor Proteins

As mentioned above, Itk associates with a plethora of adaptor molecules, most notably, SLP-76. Itk needs SLP-76 for its phosphorylation and for its localization to the T cell/APC contact site [9, 58, 63]. The interaction between Itk and SLP-76 is pivotal, as it helps to integrate Itk's functional relationship with many molecules, including LAT, PLC*γ*1, and Vav1. The interaction of Itk with SLP-76 is a complex undertaking, as it is surmised through *in vitro* studies that the interaction is a cooperative one involving SH3 domain of Itk binding to PRR of SLP-76, and then SH2 domain of Itk binding to phosphorylated tyrosine 145 of SLP-76, once it has been phosphorylated by ZAP-70 [54]. This multivalent binding of Itk to SLP-76 provides the means for Itk to associate with another adaptor molecule necessary for its activation, LAT. TCR-induced SLP-76 signalosome bridging to the LAT signalosome is mediated by the adaptor protein Gads [127]. SH3 domain of Gads binding to a PRR motif within SLP-76 separate from that of Itk indirectly connects SLP-76 to LAT when the latter becomes phosphorylated and the SH2 domain of Gads can bind that site. Although Itk does not bind Gads directly, Itk activation is therefore dependent on Gads ability to unite SLP-76 and LAT. Itk has been reported to bind LAT directly *in vitro* using GST-fusion proteins of the Itk SH2 domain [128]. This is supported by the evidence that Itk needs to be localized to the LAT signalosome for its activation [55]. Further, the association of Itk with LAT may have functional conformational consequences for Itk's activation [129]. This would imply an additional role for LAT, one that involves more than Itk localization. Therefore, the question remains whether the binding of Itk to SLP-76 or LAT is more important for Itk activation. The answer probably is both. SLP-76 and LAT are integral for the activity of a T cell, so consequently both are necessary for the activation of Itk. Lastly, Itk has also been reported to bind another adaptor molecule, Rlk/Itk binding protein (RIBP) [130, 131], which has been reported to negatively regulate Itk function, but the mechanism of this has yet to be determined.

Recently, Itk's association with SLP-76 *in vivo* has been addressed through use of an inhibiting cell-penetrating peptide [58]. This paper provided the first demonstration of the functional importance of the Itk-SH3 domain interaction with SLP-76 as it relates to Itk's localization, activation, and ultimate production of T_H_2 cytokines. This provides a mechanism of stable Itk-SH3 binding to SLP-76-PRR as a prerequisite for the subsequent binding of the Itk-SH2 domain to the phosphorylated tyrosine 145 on SLP-76, ultimately coordinating to localize and properly activate Itk [132].

### 5.4. Regulation of Cellular Adhesion

In order for T cells to initiate and maintain contact with an APC, functional adhesion through cell surface integrins are necessary. This integrin adhesion requires a functional actin cytoskeleton and also requires signaling through the TCR. Data from Jurkat T cells expressing mutant kinase domain Itk shows that Itk is integral in the activation of *β*1 integrins through the T cell receptor in an “inside-out” signaling function [133]. Further, blocking TCR-induced T cell activation through inhibition of Itk leads to a block in *β*1 and *β*2-integrin adhesion, and it affects the recruitment of LFA-1 to the site of TCR stimulation [133]. This observation was carried into primary mouse T cells by showing that TCR-induced upregulation of *β*2 integrin adhesion is also severely reduced in cells lacking Itk [134]. Thus, the TCR-induced maintenance of the T cell interaction with APC is very much dependent on Itk's kinase activity. Although the exact inter-molecular mechanism of how this cellular interaction regulated through Itk remains unknown; many Itk-associating signaling molecules involved in actin reorganization are also associated with integrin adhesion. Molecules such as PI_3_K, Vav1, PKC*θ*, and ADAP are all connected to integrin signaling, with Itk partnering PI_3_K, Vav1, and PKC*θ*. It is currently unknown whether Itk associates with ADAP, although given its extraordinary complicity with integrin signaling and its association with SLP-76, it would be a natural target to explore for Itk's regulation of integrin signaling.

### 5.5. Transcriptional Regulation

NFATc fails to translocate to the nucleus when Itk^−/−^ T cells become activated [135], as a result of diminished Ca^2+^ movement in these cells. This is an expected result because NFAT is activated through dephosphorylation by the Ca^2+^ sensitive phosphatase, calcineurin [136]. NFAT is regulated in a highly dynamic Ca^2+^ dependent manner because as intracellular Ca^2+^ levels drop, NFAT quickly becomes phosphorylated and exits from the nucleus. Consequently, for NFAT-dependent transcription to occur, quick pulses of ER-derived Ca^2+^ are not enough to sustain transcription. Rather, a prolonged surge of Ca^2+^ from extracellular sources is needed for NFAT to stay active and promote transcription [137]. Since Itk is involved in capacitative Ca^2+^ flux, Itk is also involved in prolonged NFAT transcription. NF*κ*B appears not to be defective in Itk-deficient cells, which is an interesting result as this transcription factor needs PKC to activate it, which it cannot do without sufficient DAG present [135]. DAG is needed to activate PKC leading to Erk, and ultimately, to NF*κ*B activation. Though not measured directly, DAG activity is diminished as PKC*θ*-mediated activation of Erk is reduced in Itk^−/−^ cells, and therefore one would expect that NF*κ*B activity would also be compromised. Additionally, the activation of the transcription factor AP-1 is reported to be dependent on Itk activity, as its Erk dependent ability to bind DNA is reduced [138]. Itk is also known to directly phosphorylate the transcription factor T-bet on tyrosine 525 [36, 139], which may cause the most direct means in controlling T_H_1 development. When T-bet becomes phosphorylated, it proteolytically breaksdown, leaving GATA-3 expressed, promoting a GATA-3-driven T_H_2 response. As mentioned earlier, Itk can localize to the nucleus upon activation, putatively phosphorylating T-bet at this site. However, the exact cellular location of this phosphorylation event as well as the functional significance of Itk localization to the nucleus is still unknown. The functional consequence of Itk's phosphorylation of T-bet, however, will be explained in the context of T cell differentiation.

## 6. *In Vivo* Role of Itk

At first glance, the physiological defects in cells lacking Itk would be considered modest since a deficiency in Itk has only recently been implicated in any known human disease [140]. However, mice lacking Itk display intriguing and beguiling results when it comes to cellular function. When compared to other signaling molecules involved in TCR-induced T cell signal transduction, Itk defects mirror some, though not all, defects seen with nearby molecules. The degree of Itk defects is also variable, placing Itk more as a modulator of T cell signal pathways, rather than a bridge through which all signals must pass. Table 1 summarizes the *in vivo* effects of Itk knockout mice and compares them to other knockout mice of related molecules (Lck [141], ZAP-70 [142, 143], Rlk [92], Tec [144], Itk/Rlk double knockout [92], LAT [145–147], SLP-76 [148, 149], PLC*γ*1 [150], WASp [151, 152], PKC-*θ* [153–157], Vav1 [107, 108, 158–162], ItpkB [163, 164], Gads [165], CypA [66, 166]). 

### 6.1. Development

**Figure 3:**
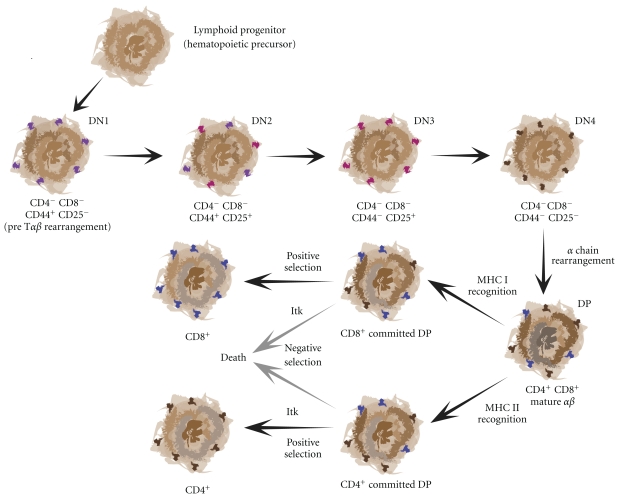
*Thymic T cell development*. Cartoon diagram of the development of a T cell from a bone marrow-derived hematopoietic progenitor to that of a mature CD4^+^ or CD8^+^ T cell capable of mounting an immune response in the periphery. Itk is necessary for both positive and negative selection, as it is able to modulate the strength of signal emanating from the T cell receptor.

Itk^−/−^ mice display decreased numbers of maturing thymocytes reduced proliferative responses and showed a profound defect in CD4^+^ T cell development Figure 3 [167]. Itk^−/−^ mice have half the number of CD4^+^ T cells, which when related to Itk molecular signaling, may be due not only to signal strength, but to signal duration as well [126]. This again signals Itk's status as a molecular rheostat. Proteins upstream and downstream of Itk, Lck, and Erk1/2, respectively, have both been shown to be important in lineage commitment [168]. Therefore, it makes sense that Itk would have defects in this area as well. However, both CD4^+^ and CD8^+^ T cells do emanate from the Itk^−/−^ thymus, but at altered ratios due to lower CD4^+^ numbers and normal CD8^+^ numbers (though this does not mean that the CD8^+^ cells are normal, as explained later). Thymic development in both fetal and adult Itk^−/−^ mice is normal when analyzed at CD4^−^CD8^−^ (double-negative, DN) and CD4^+^CD8^+^ (double-positive, DP) stages, although there is evidence that the time of progression from DN to DP stages is longer in Itk^−/−^ thymocytes when compared to wild-type cells [169]. There is a reduction seen in peripheral CD4^+^ T cells [167], which is due to an impairment in positive selection in class II MHC TCRs in Itk^−/−^ T cells [170]. There is little defect seen in positive selection using the conventional H-Y TCR system [92, 167]. Class I MHC H-Y TCRs in Itk^−/−^ T cells result in decreased efficiency of negative selection [171]. Alternatively, CD8^+^ T cell peripheral numbers are higher than controls, indicating that negative selection in the absence of Itk has failed. Both positive and negative selection need a functional calcium flux leading to the activation of Erk1/2 for positive selection [172–174] and p38 activation for negative selection [175]. Itk^−/−^ T cells do not possess an optimal calcium flux and fail to activate Erk1/2, but p38 activation is still intact. Furthermore, in the paper by Huang et al., positive selection is affected while negative selection is spared when Itk becomes inactive due to the lack of secondary messenger IP_4_ [49]. These data indicate that Itk would have a more profound impact on positive selection rather than negative selection. This is confirmed when in the absence of Itk, the deletion of self-reactive thymocytes under strong stimulus conditions is impaired, but not under weak stimulus conditions. Only when Itk-deficient thymocytes continue to progress to a later stage of development do they succumb to negative selection signals [170].

Although the total numbers of CD8^+^ T cells from Itk-deficient mice appear to be normal, conventional CD8^+^ T cells are severely diminished. Instead, an innate or memory cellular phenotype exists in these mice causing the CD8^+^ T cell numbers to appear normal [176, 177]. CD8^+^ thymocytes from Itk^−/−^ mice have a memory cell surface phenotype (CD44^hi^, CD62L^hi^, CD122^hi^) and include natural killer cell markers, such as NK1.1. Additionally, Itk^−/−^ CD8^+^ T cells spontaneously express IFN*γex vivo* and upregulate antiapoptosis genes such as BCL-X_L_ upon IL-15 stimulation, which is similar to memory cells. Furthermore, independent thymic development of CD8^+^ T cells in Itk^−/−^  mice is observed regardless of the type of cell presenting MHC class I, whereas conventional CD8^+^ thymic derived T cells are only selected through interaction with thymic epithelial MHC class I presentation. Finally, Itk^−/−^  CD8^+^ T cells are dependent on the presence of IL-15 for their survival, again similar to a memory cell phenotype [178]. The work to describe the existence of these innate CD8^+^ T cells emanating from Itk^−/−^ mice was done in the Schwartzberg lab where they showed these cells arise in Itk^−/−^ fetal thymic organ cultures [176]. Furthermore, a molecular marker for memory CD8^+^ T cells, eomesodermin, is upregulated in the absence of Itk [177]. This is interesting because eomesodermin is known to upregulate the expression of both the memory cell marker CD122 and the expression of IFN*γ*  [179]. Finally, the rise of these innate-like CD8^+^ T cells is dependent on the adaptor SAP, like NKT cells [180]. Therefore, Itk-deficient mice provide an interesting insight into the development of CD8^+^ memory T cells.

### 6.2. Differentiation

**Figure 4:**
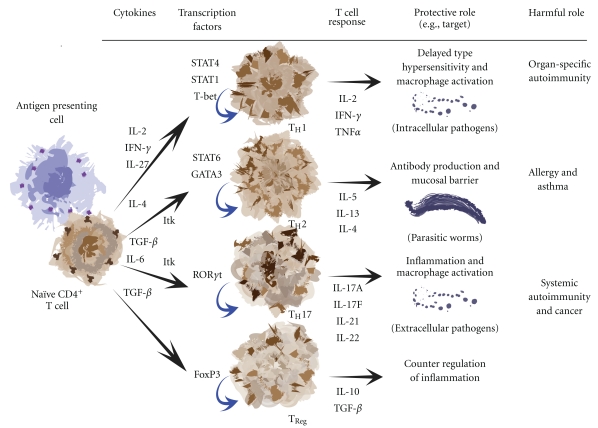
*T cell differentiation and their responses to pathogens*. Cartoon diagram displaying the interaction of a naïve CD4^+^ T cell with a dendritic cell APC leading to differentiation into T helper subsets. The development of these subsets allows for protective immunity against specific pathogens. Itk is needed to promote T_H_2 and T_H_17 differentiation.

T helper cell differentiation is a necessary and crucial developmental process that selectively hones the T cell response towards certain pathogens (Figure 4). Initially, these differentiated cells were divided into two types, T helper 1 (T_H_1) and T helper 2 (T_H_2). This delineation is evolving to include T helper 17 (T_H_17) and regulatory T cells (T_Reg_) as well.

During T_H_1 differentiation, IL-12, IL-27, or IFN*γ* signaling through STAT4 and STAT1 induces the expression of the transcription factor T-bet, which can directly drive T_H_1 development by increasing IFN*γ* production and inhibiting IL-4 production. T_H_2 differentiation, by contrast, is dependent on the IL-4-induced activation of STAT6 leading to the activity of the transcription factor GATA-3, causing an increase in IL-4 production and a decrease in IFN*γ* production. T_H_2 differentiation was recently described to occur in two steps when examined in mice expressing an IL-4 dual reporter gene [181]. The first is the T_H_2 competent stage where cells initially upregulate IL-4 and GATA-3 gene expression. The second is the T_H_2 effector stage where cells support the production and release of T_H_2 cytokines necessary for effector function. This two-step T_H_2 differentiation model was extended by use of an IL-4-GFP reporter system crossed into Itk-deficient mice [182]. In these studies, it was found that in the absence of Itk, T_H_2 differentiation could proceed normally, but when these differentiated cells needed to produce and secrete T_H_2 effector cytokines, they could not do so even with repeated stimulation. Additionally, under non-skewing conditions (no exogenous IL-4 added *ex vivo* to the developing cultures to promote T_H_2 differentiation) Itk^−/−^ cells display defects in T_H_2 competent cells, as they preferentially develop into IFN*γ* producing T_H_1 cells [36]. This is an interesting result, as IL-2 receptor signaling is necessary for IL-4 transcription [183], and since Itk^−/−^ mice fail to produce substantial amounts of IL-2 upon stimulation, IL-4 production could be impaired due to the lack of autocrine IL-2 signaling necessary to stabilize transcription factor access to the *Il4* gene leading to T_H_2 differentiation [184]. Furthermore, the relative ratios of Itk and Rlk during T helper cell differentiation can also be useful in determining which pathway the cell will take. As mentioned earlier, naïve CD4^+^ T cells express 3–5 times more Itk than Rlk when measured by qRT-PCR. When the T cell becomes activated through its T cell receptor, Rlk becomes downregulated, leaving 100 times more Itk than Rlk present [36]. While in this activated state exposure to cytokines secreted by resident non-T cell leukocytes provides a skewing environment that drives T helper cell differentiation, which is greatly influenced by the increased ratio of Itk.

T_H_1 and T_H_2 effector cells are also characterized by the expression of two transcription factors, T-bet in T_H_1 cells, GATA-3 in T_H_2 cells. T-bet is required for the differentiation of naïve T helper cells into T_H_1 cells and expression of IFN*γ*  [185], while GATA-3 regulates chromatin remodeling at the locus that encodes the cytokines IL-4, IL-5, and IL-13, and consequently promotes T_H_2 differentiation [186, 187]. Itk plays an active role in the expression of these transcription factors, as it phosphorylates T-bet on tyrosine 525 [36, 70]. Phosphorylation of T-bet leads to its proteolytic breakdown, leaving only GATA-3 to be expressed, and therefore, a T_H_2 effector cell differentiated phenotype.

An intriguing question asked recently is whether Itk is involved in the regulation of T_H_17 cellular function. T_H_17 cells normally mediate the host defensive response to extracellular bacterial infections and are reportedly involved in the pathogenesis of autoimmune diseases through the overexpression of proinflammatory cytokines. The signature cytokines of T_H_1 and T_H_2 cells, IFN*γ* and IL-4 respectively, have both been shown to inhibit T_H_17 differentiation [188, 189]. Markedly, while IFN*γ* normally upregulates T-bet, the expression of T-bet is significantly lower in T_H_17 cells [190, 191]. Further, the promotion of T_H_17 development leads to the pathogenesis of experimental autoimmune encephalomyelitis [192]. The T_H_17 signature cytokine IL-17 is overexpressed in the airways of asthmatics where it induces the production of IL-8 chemoattractant needed by neutrophils, which are phagocytic granulocytes involved in eliminating bacterial infections [193]. These findings draw a connection between Itk, T_H_17 cells, asthma, and autoimmunity through the transcription factor T-bet. Since Itk phosphorylates and affects the downregulation of T-bet, it would be expected that Itk promotes the development of T_H_17 cells. This is indeed the case as shown recently by Gomez-Rodriguez et al. [194]. It was found in this work that Itk^−/−^ T cells produce less IL-17A than their wild-type counterparts through a reduction in *IL17a* transcripts, though interestingly enough, *IL17f* transcript production was intact as was the expression of the transcription factor that regulates T_H_17 differentiation, ROR*γ*t. This is a fascinating result that implies T_H_17 differentiated T cells can be further subdivided into functional subsets [195]. Lastly, since the presence of Itk helps to promote IL-17A production, the recent finding that IL-17A provides a protective function in a colitis model system suggests that Itk too can be important in the mediation of intestinal inflammation [196].

Another re-emerging set of differentiated CD4^+^ T cells that have garnered lots of interest lately are regulatory T cells, or T_Reg_ cells. T_Reg_ cells have a critical function in the regulation of peripheral tolerance. It may be that T_Reg_ and T_H_17 cells are reciprocally regulated through the activation and deactivation of transcription factors, much like inverse the relationship between T_H_1 and T_H_2 cells [197]. The main cytokine produced by T_Reg_ cells is IL-10. This cytokine inhibits the expression of many proinflammatory cytokines and chemokines, helping to decrease the number of circulating leukocytes from entering the inflamed tissue. Notably, another significant defect in Itk-deficient T cells is the failure to produce IL-10 in response to activation [138]. This was previously interpreted to be part of a diminished T_H_2 function. However, given what is now known about the T_Reg_ signature cytokine being IL-10, it would be interesting to determine the function of these T_Reg_ cells in the absence of Itk. Speculatively, Itk^−/−^ T cells could have a diminished T_Reg_ response leading to hyperinflammatory conditions.

The regulation of the T lymphocyte cytoskeleton by Itk is through an intricate dance between two additional cytoskeletal molecules, Vav1 and WASp. Although Itk may not be integral in the phosphorylation of WASp [118], it is intimately involved in locating WASp to the immunological synapse thereby affecting WASp activity [105]. Recently, it has been discovered that WASp is important in the development and function of T_Reg_ cells, in both mice and in humans [198–201]. These studies confirm that WASp-deficiency curbed the differentiation of these cells, as well as abrogated their suppressive cellular activity, such as suppressing the production of TGF*β* and IL-10. Given the relationship between Itk adaptor function and the localization of WASp necessary for its activity, a similar interaction in T_Reg_ may indicate that Itk may very well be involved in the management of T_Reg_ activity, thus affecting autoimmune disorders.

### 6.3. Cytokine Production

IL-2 production in T cells lacking Itk is decreased in response to stimulation through the T cell receptor, which affects these cells ability to proliferate [92, 167, 202]. Exogenous IL-2 enables these cells to overcome this proliferative defect, a response that originally led to the name of this kinase [27, 92].

Activation of naïve CD4^+^ lymphocytes causes their differentiation into either T_H_1 or T_H_2 effector cells, which produce distinct sets of cytokines. T_H_1 cells preferentially produce IFN*γ*, TNF*α*, and IL-2, while T_H_2 cells primarily produce IL-4, IL-5, and IL-13. The balance between these two sets of CD4^+^ effector cells allows for the tuning of the immune response to the state of infection. Conversely, when this balance is upset, self-reactive disease states can ensue. An excess of T_H_1 cells can lead to autoimmune states, while an excess of T_H_2 cells can lead to hypersensitivity. Therefore, T helper cell differentiation and cytokine expression regulation is of paramount importance for an adequate immune response. Itk^−/−^ mice display a peculiar cytokine production profile, as the CD4^+^ T cells emanating from these mice produce relatively normal levels of T_H_1 cytokines (e.g., TNF*α* and IFN*γ*), while T_H_2 cytokine expression (IL-4, IL-5, and IL-13) is diminished [135, 203]. IL-4 expression can be rescued by the retroviral expression of Itk in Itk-deficient cells, indicating that Itk is the sole reason for the defect in these cells and not something due to the development of these cells in the absence of Itk [135]. Since Itk^−/−^ cells have an impaired NFAT transcriptional activity, it is therefore expected that IL-4 and IL-13 cytokine production would be hindered as both of those cytokines are dependent upon NFAT activity [204].

### 6.4. Pathogen Clearance

During the course of a viral infection CD8^+^ T cells differentiate into activated killer cytotoxic T lymphocytes (CTL) whose main functions are the lysis of infected cells and the secretion of antiviral cytokines, such as IFN*γ* and TNF*α*. CD8^+^ T cell function in cells lacking Itk has yielded perplexing results, as Itk^−/−^ mice were initially thought to have only a mild defect in their response to viral infection [205]. When infected with either lymphocytic choriomeningitis virus (LCMV), vaccinia virus (VV), or vesicular stomatitis virus (VSV), CTL responses were reduced two to six-fold. However, the kinetics of viral clearance was the same in Itk^−/−^ mice as in wild-type mice. Further, in the case of anti-VSV-antibodies, the serum titers of these antibodies were normal in Itk^−/−^ mice when compared to wild-type mice when each had been infected with VSV. A very interesting recent finding is the determination that Itk can affect the replication of human immunodeficiency virus (HIV). Through use of Itk-specific siRNA or by using an Itk chemical inhibitor, production of HIV protein p24, actin-dependent HIV viral entry, and HIV transcription were all reduced [206]. Although this research does not imply Itk in the direct clearance of HIV virally infected cells by CTLs, it does show that by inhibiting Itk activity, one can ameliorate a viral infection.

T helper cell differentiation is crucial for the clearance of pathogens. T_H_1 cells are important in promoting responses to clear intracellular pathogens, whereas T_H_2 cells support productive humoral immune responses against extracellular pathogens. Itk^−/−^ mice display an increased susceptibility to parasitic infection, as displayed in their inability to clear the intracellular pathogen *Toxoplasma gondii* (*T. gondii*) [92], a parasite that normally induces a protective T_H_1 response in wild-type mice. This is odd because *T. gondii *requires a fast and robust IFN*γ* response by the host to prevent lethality, which Itk^−/−^  mice produce. This may be due to the Itk^−/−^ mouse strain used for these studies since there are differences in T_H_1 and T_H_2 differentiation among mouse strains [207, 208]. During a *Leishmania major *(*L. major*) infection, wild-type C57Bl/6 mice are able to produce T_H_1 cytokines resulting in the clearance of the parasite, whereas wild-type BALB/c mice primarily produce T_H_2 cytokines, which causes them to succumb to the infection. During an infection that requires a T_H_1 response by the host to clear the infection, Itk^−/−^ mice on C57Bl/6 background elicit a protective T_H_1 response [135]. However, BALB/c mice deficient for Itk are unable to normally produce a T_H_1 response, and could not produce a T_H_2 response due to the absence of Itk, resulting in the inability of the mice to clear the infection. The discrepancy between these results may be because *T. gondii* infection requires a rapid T_H_1 response, while *L. major* can be cleared by a slow, less robust response. Therefore, the kinetics of infection and response may dictate the lethality of the pathogenic infection.

In an attempt to address this issue, the Fowell group backcrossed the Itk^−/−^ mice on IL-4-GFP reporter mouse background [209] to examine an IL-4 producing T_H_2 response during *L. major* infection. IL-4 producing CD4^+^ T cells were present at the site of infection, indicating that differentiation and migration to site of infection were not a problem. However, the amount of IL-4 produced, particularly upon restimulation, was insufficient in the Itk^−/−^ mice when compared to the wild-type mice [182]. This elegantly determines that Itk is not necessarily required for differentiation, but is required in the production of this cytokine.

Meanwhile, T_H_2 effector function for protective immunity of extracellular pathogens is more clear. Infection with *Nippostrongylus brasilinensis* (*N. brasilinensis*) causes a protective T_H_2 response in BALB/c mice, while Itk^−/−^ BALB/c mice were unable to clear the nematode due to a suboptimal T_H_2 response [135]. Similarly, Itk^−/−^ mice on a C57Bl/6 strain background were unable to mount a protective T_H_2 response to the helminth *Schistosoma mansoni* (*S. mansoni*) due to a severe reduction in T_H_2 cytokine production of IL-4 and IL-5 [138].

### 6.5. Allergy Induced Bronchial Asthma

The connection between Itk and allergic responses was first made in a clinical study on patients with atopic dermatitis, a disease state characterized by an itchy rash and inflammation caused by an excess of T_H_2 cell response [210]. There is an increased incidence of Itk expression in these patients correlating to the severity of the disease state. This corresponds well with elevated Itk expression levels in mouse T_H_2 cells [36]. Furthermore, the linkage between atopy and Itk can be traced genetically to the Itk genomic locus as there are single nucleotide polymorphisms at this locus in atopic patients [211]. T_H_2 cellular responses are involved in the pathology of allergic asthma, as the number of T_H_2 cells recruited to the lungs (an immune privileged site) is increased, as well as the expression of T_H_2 cytokines and the consequent movement of responding cells to the lungs resulting in increased inflammation and mucus production [212, 213]. Itk's first direct molecular and cellular correlation to asthma was made by Mueller and August where they induced airway hyperresponsiveness in mice and found that Itk^−/−^ mice displayed a reduced number of T cells and eosinophils infiltrating the lungs when compared to similarly treated wild-type mice [203]. This finding included a reduction in lung inflammation, diminished mucus production, and reduction in the T_H_2 cytokine expression of IL-5 and IL-13. This was later extended by Ferrara et al. in the finding that Itk^−/−^ mice display a diminished tracheal response to allergen challenge using the same airway hyperresponsiveness model system [214]. Mast cells degranulation was also found to be impaired in Itk^−/−^ mice in the same model system [215]. On a related issue, mice lacking the transcription factor T-bet, which Itk normally phosphorylates to promote T-bet's degradation, spontaneously develop airway hyperresponsiveness and display enhanced inflammation and an increase in secreted T_H_2 cytokines [216]. To support this idea, the August lab recently published work that the Itk kinase domain activity is necessary for the development of allergic asthma [217]. Interestingly, T cells from these Itk kinase domain lacking mice displayed a profound defect in cytokine mediated T cell migration. Given these results, competitive inhibitors that selectively target Itk would be of beneficial therapeutic value in the treatment of asthma. In fact, one study has shown that by inhibiting Itk kinase activity, lung inflammation in mice can be ameliorated [91].

### 6.6. Apoptosis

Activation induced cell death (AICD) is the process by which cells are instructed to commit suicide, or apoptose, in response to an extracellular signal. This process is genetically linked as most cells contain signaling pathways always on the ready for the signal to apoptose. Functionally, this process is crucial in the life of a lymphocyte, as stimulation-induced clonal expansion leads to an enormous number of cells generated to combat an infection, the clearance of the invaders leads to many cells that are no longer needed. Apoptosis serves as a means to eliminate these extra cells, which (if no more pathogen is present) can possibly be destructive. During instructive thymic development, T lymphocytes that give too robust a signal to self-antigen during positive selection are signaled to apoptose in order to limit self-reactive T cells from entering the periphery. The apoptotic elimination of clonally expanded T cells after an infection is also necessary to prevent autoimmunity, as an abundance of no longer needed cells could cause destruction of tissue with similar epitopes [218, 219]. Itk is keenly involved in the apoptosis of T cells. Itk^−/−^ T cells have a decreased susceptibility to CD3-induced apoptosis [92]. Thymocyte deletion, particularly that of double-positive T cells, is also defective in cells lacking Itk [171]. Furthermore, Itk is involved with the expression of Fas-ligand as well as the interaction between Fas-receptor and its downstream cell death induction molecules [202]. Itk, however, has not been shown to be involved in anti-Fas engagement leading to apoptosis. Whether Itk influences the apoptosis of the large population of CD8^+^ memory-like T cells remains to be seen.

### 6.7. In Vivo Comparison to Other Signaling Molecules

Itk-deficient cells display many commonalities with cells deficient in other signaling molecules within the TCR-induced signalosome (Table 1). LAT^−/−^ mice do not develop mature T cells, although transgenic point mutant Y136F mice (the LAT tyrosine motif that PLC*γ*1 binds to [220]) have a similar phenotype to that of Itk^−/−^ mice [145, 146]. These mice have impaired Ca^2+^ mobilization, T_H_2 cytokine production, and increased amounts of IgE in the serum. JNK^−/−^ mice are also incapable of clearing a *L. major *infection, indicating that Itk could also act through the JNK pathway. These JNK-deficient mice have a reduced IL-4 yield, yet are able to produce IFN*γ* when stimulated *in vitro* under nonskewing conditions much like that of Itk^−/−^ mice. Mice deficient in cyclophilin A (CypA) spontaneously develop allergic disease marked by increases in IgE antibody titer in serum. These mice also have an increased infiltration of mast cells and eosinophils into the lungs [66]. T_H_2 cells emanating from these mice are hypersensitive to TCR stimulation, and although Itk activity has not been directly assessed in these cells, given CypA repressive function on Itk, it would be assumed that Itk could be hyperactive in these cells. Vav1 and PKC*θ* deficient T cells have a decrease in IL-2 production, and consequently, IL-4 production as well [154, 155, 161, 162, 221]. Downstream, these cells are defective in Ca^2+^ mobilization leading to a defect in NFAT transcriptional activation, these cells are similar to Itk^−/−^ cells as they are defective in AP-1 transcription factor activation, and these cells are defective in NF*κ*B activity. Additionally, mice defective for the expression of NFAT1 have a decreased T_H_2 cytokine expression profile [204]. All of these molecules provide evidence linking the involvement of Itk in their cellular function.

### 6.8. Itk Function in Unconventional and Non-T Cells

Although much of the research on Itk has been produced in conventional *αβ* T cells, Itk is expressed in natural killer (NK) cells, natural killer T (NKT) cells, and mast cells. natural killer cells are lymphocytes that can eliminate virally infected cells or transformed cells, independent of an antigen stimulus as in CTLs. The NK cellular response in Itk^−/−^ or in Itk^−/−^Rlk^−/−^ double knockout cells was first reported to be normal, as interpreted from the robust response to LCMV infection in these mice [35, 205]. However, recently it was determined that Itk both positively regulates NK cell cytotoxicity in response to FcR stimulation and negatively regulates NK cell cytotoxicity upon NKG2D receptor activation [222]. This indicates a more complex role for Itk in NK function, one that deserves further exploration. Mast cells are tissue-resident granulocytes that are the effector cells in immediate hypersensitivity reactions such as allergic rhinitis. The role of Itk in mast cells is blurred by the presence of Btk, where Btk seems to be the dominant Tec kinase in these cells. Itk-deficient mast cells elicit little difficulty in producing a cellular response, while Btk-deficient mast cells have much difficulty doing the same in most experimental cases of hypersensitivity [223]. However, in the specific context of an acute allergic response in the lung, Mast cell degranulation in Itk^−/−^ mice was significantly reduced, irrespective of late phase T_H_2-dependent inflammatory defects [215]. This is due to saturating levels of IgE occupancy of Fc*ε*RI in these mice, which may interfere with the degranulation response of newly secreted IgE [224]. NKT cells are a rare heterogenous subset of T cells that are important for the initiation and regulation of an immune response through the ability to immediately secrete large quantities of cytokines, particularly IFN*γ* and IL-4. Itk has recently been shown to be important for the development and the homeostasis of NKT cells [225, 226]. Interestingly, not only are NKT cell numbers reduced, but the continued homeostasis of these cells is dependent on Itk as NKT cells are diminished even further in Itk^−/−^ mice as they age when compared to strain-matched control mice. Itk is involved in the production of both IFN*γ* and IL-4 in NKT cells [227], as Itk-deficient NKT cells fail to produce either cytokine thus making the cells functionally defective. This is an interesting finding because this is different from the T_H_2-skewed phenotype seen in T cells from Itk-deficient mice where only IL-4 production is affected. Lately it was found that although Itk is necessary for the development of *αβ* T cells, it was not dispensible for *γδ* T cells [228]. Itk may act as a negative regulator for these cells, as their numbers were increased with respect to wild-type mice, particularly within the *γδ* NKT subset. These cells are activated and provide T cell help as seen by elevated levels of IgE [228, 229].

## 7. Conclusions

Itk can be an ideal therapeutic target for the regulation of T_H_2 cell-mediated diseases. Since Itk is the Tec kinase primarily expressed in T_H_2 cells, the selective inhibition of its activity could affect T_H_2 cell function, including cytokine expression, without affecting immune responses against other infections, such as viral or T_H_1 cell-mediated pathogens. As such, Itk is a TCR proximal rheostat serving as a critical molecule that affects signaling and development in subtle ways, helping to evaluate the strength of the TCR signal in order to produce a T cell response that is appropriate for the presented situation.

## Figures and Tables

**Table 1:** Comparison of phenotypes between TCR-inducible signaling molecules. Table depicting developmental, biochemical, and phenotypic defects observed in mice deficient for the indicated gene in the first column. A “Yes” response denotes confirmation of the defect, while “N.A.” indicates “not applicable” and “N.D.” indicates “not determined”. References for the defects are listed in the last column.

	Developmental defects		Biochemical defects								*In vivo *phenotype defects					Ref.
KO mouse	Stage pre- TCR	Affected	PLC*γ*l phos	Ca^++^ flux	Erk phos	NFAT activ	IL-2 prod	Proliferation	Actin polym	Adhesion	Activated phenotype	AICD	T_H_2 response	Pathogen clearance	Eosinophilia	
Itk	Mild	DP-SP	Yes	Mild	Yes	Yes	Yes	Mild	Yes	Yes	Yes	Yes	Yes	Yes	Mild	[36, 92, 167, 170, 202, 204, 205]
RIk	No	No	No	No	No	No	Mild	No	No	No	N.D.	N.D.	N.D.	N.D.	N.D.	[92]
Tec	No	No	N.D.	N.D.	N.D.	N.D.	No	No	N.D.	N.D.	N.D.	N.D.	N.D.	N.D.	N.D.	[144]
Itk/Rlk	Yes	DP-SP	Yes	Yes	Yes	Yes	Yes	Yes	Yes	Yes	Yes	Yes	No	No	N.D.	[92]
Lck	Yes	DN1	Yes	Yes	Yes	Yes	Yes	Yes	Yes	N.D.	N.A.	N.A.	N.A.	N.A.	N.A.	[141]
ZAP-70	Yes	DN3-DN4	Yes	Yes	Yes	Yes	Yes	Yes	Yes	N.D.	N.A.	N.A.	N.A.	N.A.	N.A.	[142, 143]
LAT^ Y136F^	Yes	DN3-DN4	Yes	Yes	No	Yes	Yes	Yes	Yes	Yes	Yes	Yes	Increase	N.D.	Yes	[145–147]
SLP-76	Yes	DN1	Yes	Yes	Yes	Yes	N.D.	Yes	Yes	N.D.	N.A.	N.A.	N.A.	N.A.	N.A.	[148, 149]
Vav1	Mild	DP-SP	Yes	Yes	Yes	Yes	Yes	Yes	Yes	Yes	N.D.	N.D.	Yes	N.D.	N.D.	[107, 108, 158–162]
ItpkB	Yes	DP-SP	Yes	No	Yes	N.D.	N.D.	N.D.	N.D.	N.D.	N.D.	N.D.	N.D.	N.D.	N.D.	[49, 163, 164]
Gads	Yes	DN3-DN4	Yes	Yes	Yes	Yes	Yes	Yes	N.D.	N.D.	N.D.	N.D.	N.D.	N.D.	N.D.	[165]
PLC*γ*l	Mild	DP-SP	Yes	Yes	N.D.	Yes	Yes	Yes	N.D.	N.D.	N.D.	N.D.	N.D.	N.D.	N.D.	[150]
WASp	Mild	No	N.D.	Mild	No	Yes	Yes	Yes	Yes	No	Yes	N.D.	Yes	N.D.	N.D.	[151, 152]
PKC*θ*	No	No	Yes	Yes	No	Yes	Yes	Yes	Yes	N.D.	N.D.	Yes	Yes	Yes	N.D.	[153–157]
CypA	No	No	No	No	No	Yes	Yes	Yes	N.D.	N.D.	N.D.	N.D.	Yes	N.D.	N.D.	[66, 166]
